# Does the Medical College Admission Test (MCAT) predict licensing examination performance in the Canadian context?

**Published:** 2019-03-13

**Authors:** Maitreyi Raman, Sara Lukmanji, Ian Walker, Douglas Myhre, Sylvain Coderre, Kevin McLaughlin

**Affiliations:** 1 University of Calgary, Alberta, Canada

## Abstract

**Background:**

Research on the predictive validity of the Medical College Admissions Test (MCAT) on licensing examination performance is varied in its conclusions, with only a few studies examining this relationship in a Canadian context. We assessed the predictive validity of the MCAT on successful performance on the Medical Council of Canada Qualifying Examination (MCCQE) Part 1 by students attending the Cumming School of Medicine.

**Methods:**

Prospective data were collected on MCAT score and sub-section scores, MCCQE decision, multiple mini interview (MMI) performance, gender, and age. The cohort was divided into a derivation cohort (2013 and 2014) and validation cohort (2015 and 2016). Students were dichotomized into pass or fail on MCCQE. Multiple logistic regression in which our dependent variable was MCCQE Part I examination success at the first attempt was used, and potential explanatory variables were age, gender, MCAT total score, and sub-scores for the biological sciences (MCAT-BS), physical sciences, and verbal reasoning, GPA, and MMI ratings.

**Results:**

For the derivation cohort MCAT-BS was associated with success on the MCCQE Part I. The odds ratio for this association of 1.37 (95% confidence interval [1.01, 1.85], p = 0.04). When we applied the MCAT-BS to our validation cohort the odds ratio of MCCQE Part I examination success was 1.42 [1.10, 1.83], p = 0.007) and the area under the ROC curve was 0.66 [0.54, 0.79]).

**Conclusion:**

The MCAT-BS predicted successful performance on the MCCQE Part 1 Examination in the Canadian setting.

## Introduction

Medical school admissions committees are faced with the challenge of selecting students for admission from a large pool of applicants. The decisions made throughout this selection process have important implications on society as they impact the ability of the health care system to meet the needs of the population. For example, ensuring that prospective students have equitable access to medical school admission, while also possessing the necessary academic and non-academic skills to succeed in a career in medicine, is important to producing physicians that reflect the diversity of the population, are effectively able to serve their patients, and advance scientific discovery.^1^ Therefore, it is important that medical school admissions committees evaluate applicants in a reliable and valid manner in order to select students that will succeed in their medical education, subsequent careers as physicians, and embody the mission of their medical schools.

In Canada, the admissions selections process varies between medical schools, but generally involves multiple assessment methods that evaluate both academic and non-academic traits. The tools utilized to measure academic abilities vary between medical schools and may include all measures or a selection of Grade Point Average (GPA), the Medical College Admissions Test (MCAT) scores, and holistic file reviews of academic achievement. To assess academic preparation, MCAT is a commonly used tool across North America.

The widespread use of the MCAT is largely due to its ability to facilitate reliable comparison between applicants from diverse backgrounds arising from its quantitative and standardized nature.^2^^,^^3^ There have also been several studies that demonstrate that the MCAT can predict future performance on licensing examinations. A systematic review and meta-analysis of 19 studies found that the MCAT total score has moderate to high predictive validity for success on the United States Medical Licensing Examination (USMLE) Step 1, although this relationship is not as pronounced for the USMLE Steps 2 and 3 which are focused on assessing clinical knowledge and skills compared with pre-clinical basic science knowledge tested in Step 1.^4^ This systematic review also found that the biological sciences subsection of the MCAT was the best predictor of USMLE Step 1 scores.^4^ More recent studies have shown that both MCAT total score and MCAT biological sciences sub-score were predictive of performance on the USMLE Step 1.^5^^,^^6^ However, a weak or insignificant relationship between the MCAT and licensing examination scores or board certification has also been demonstrated.^7^^,^^8^ The predictive validity of the MCAT, and subsections of the MCAT, on licensing examination scores has also been shown to be influenced by factors such as English language learner status and ethnicity.^9^^,^^10^^,^^11^

Not only is the literature examining the MCAT and performance on licensing examinations somewhat varied in its conclusions, most of it is also based on American populations, with only a few studies examining this relationship in a Canadian context.^12^^,^^13^^,14^ Successful performance on the Medical Council of Canada Qualifying Examinations (MCCQE) is required for independent clinical practice. Part 1 of the MCCQE examination is a computer-based test that assesses the competence of candidates who have obtained their medical degree for entry into postgraduate training programs. Part 2 of the MCCQE consists of a series of clinical stations to assess the knowledge, skills, and attitudes prior to entry into independent practice. In the Canadian context, the predictive validity of the MCAT on performance of MCCQE examination has been examined, with identification of the verbal reasoning and biological sciences subsections correlated with Part I scores.^12^^,^^13^

Prior to the release of the MCAT 2015, the admissions selections process at The Cumming School of Medicine (CSM) at the University of Calgary considered the following scores in its admissions selections process: GPA, MCAT verbal reasoning (MCAT-VR) sub-score, holistic file review, and Multiple Mini Interview (MMI). The MCAT-VR sub-score is assigned 10% weighting to the final admissions score based on literature supporting its predictive validity. Although an assigned weighting to the total MCAT score or other sub-scores is not provided, the file reviewers are encouraged to consider all aspects of academic and non-academic performance when assigning a holistic file review score and are able to see all MCAT components.

The relationship between MCAT scores and performance on medical licensing exams warrants further investigation in a Canadian context. The objective of our study was to assess the predictive validity of the MCAT on successful performance on the MCCQE Part 1 by students attending the CSM to better inform the admissions selection process there and in other similar medical schools.

## Methods

This study was granted ethical approval by the Conjoint Health Research Ethics Board at the University of Calgary.

### Participants

Our participants were all students at the CSM who completed the MCCQE Part 1 between the years 2013-2016 inclusive.

### Data sources

The data used for this study were collected prospectively as part of our admission selection process. These data included scores from GPA, MCAT total score and sub-scores (biologic sciences, physical sciences, verbal reasoning), and the MMI. We also noted the gender and age of applicants.

### Procedures

We used a retrospective case-control study design to identify variables collected at the time of medical school admission that associate with pass/fail performance on the MCCQE Part 1 exam. We divided our dataset into derivation and validation cohorts to allow us to determine whether any associations between admissions data and MCCQE Part 1 exam were consistent.

In 2015, the Medical Council of Canada (MCC) appointed a standard-setting panel comprised of 17 Canadian physicians to identify a new minimum pass level (427) for the MCCQE Part 1. In doing so, they also developed a new scale for scoring the examination – a numeric result between 50 and 900 (mean 500, SD 100), which would be used during all future examination sittings. For the validation cohort (2015 and 2016) we dichotomized students into pass vs. fail depending upon whether their score on the MCCQE Part I examination was ≥ 427 or < 427, respectively, and for the derivation cohort (2013 and 2014) cohorts we dichotomized students into pass vs. fail using a cut-off of 440 (which equates to 427 on the revised scale).

### Statistical analysis

We divided our dataset into a derivation cohort (282 students from the graduating classes of 2013 and 2014) and a validation cohort (269 students from the graduating classes of 2015 and 2016). In our derivation cohort we used multiple logistic regression in which our dependent variable was MCCQE Part I examination success at the first attempt and our potential explanatory variables were age, gender, MCAT sub-scores for the biological sciences (MCAT-BS), physical sciences (MCAT-PS), and verbal reasoning (MCAT-VR), GPA, and MMI ratings. We also considered two-way interactions between our explanatory variables and performed backward elimination in our regression model, beginning with the interaction terms. From the results of our logistic regression we then generated probability of MCCQE Part I examination failure using the equation: probability = e^a+bx^ /1+e^a+bx^. To evaluate the predictive ability of our explanatory variables we performed receiver operating characteristic (ROC) analysis where our outcome was MCCQE Part I examination success. Having identified the predictors of MCCQE Part I examination success in our derivation cohort, we then evaluated the predictive performance in our validation cohort. We used STATA^®^ Version 11.0 (StataCorp, College Station, Texas) for our statistical analyses.

## Results

### Derivation cohort

**Figure 1:**
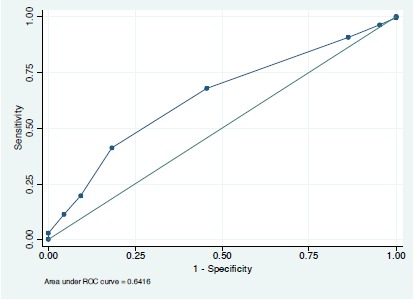
Area under the ROC curve for MCAT-BS as a predictor of success on the MCCQE Part I examination in the derivation cohort

**Figure 2:**
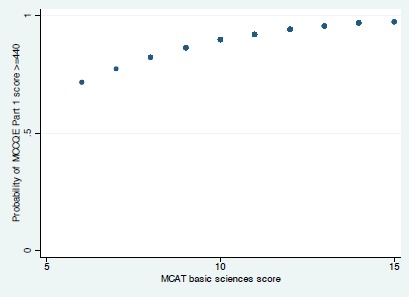
The association between MCAT-BS and the probability of success on the MCCQE Part I examination in the derivation cohort

For the derivation cohort (students from the classes of 2013 and 2014), there were no statistically significant interactions and a single variable, MCAT-BS, was associated with success on the MCCQE Part I examination. The odds ratio for this association of 1.37 (95% confidence interval [1.01, 1.85], p = 0.04) implies that the odds of examination success increases by 37% for each point increase in the MCAT-BS score. In terms of predicting MCCQE Part I examination success, the area under the ROC curve was 0.64 [0.53, 0.76] (Figure 1). Figure 2 shows the relationship between MCAT-BS score and the probability of success on the MCCQE Part I examination.

### Validation cohort

**Figure 3:**
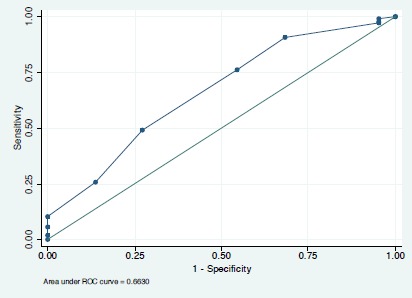
Area under the ROC curve for MCAT-BS as a predictor of success on the MCCQE Part I examination in the validation cohort

When we applied the pre-clerkship MCAT-BS to our validation cohort the odds ratio of MCCQE Part I examination success was 1.42 [1.10, 1.83], p = 0.007) and the area under the ROC curve was 0.66 [0.54, 0.79]) (Figure 3). Figure 4 shows the relationship between MCAT-BS score and the probability of success on the MCCQE Part I examination for the validation cohort.

## Discussion

In our prospective longitudinal study, we have shown that the MCAT-BS predicted successful performance on the MCCQE Part 1 Examination. As the admissions selection process pre-assigned 10% weight to the file score, the MCAT-VR sub-score could not be assessed as predictive of outcome. Consequently, our study sample had higher MCAT-VR scores and may not have included a wide enough range of MCAT-VR scores to establish the predictive validity of this MCAT subsection.

**Figure:**
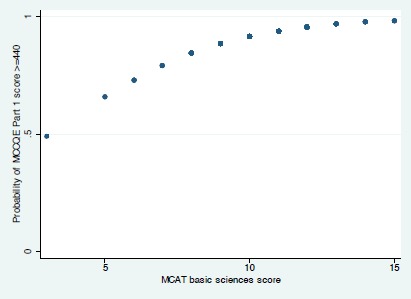
Figure 4. The association between MCAT-BS and the probability of success on the MCCQE Part I examination in the validation cohort

Demographic variables (age, gender) and other measures of academic performance (GPA, MMI score) were not shown to predict successful MCCQE Part 1 performance. Our findings compare with previously published studies although others have shown that MCAT total scores, or subsection scores other than the MCAT-BS section, were predictive of licensing exam success.^4^^,^^6^

Potential explanations to account for the findings and those of previously published reports may include differences in institutional curricular content in the basic sciences, and differences between our cohort of students and those studied in these reports. For example, one study found MCAT total score was the best predictor of USMLE step 1 performance, however their sample was an exclusively Asian and Pacific Islander population.^7^ Others have demonstrated that MCAT total score was weakly associated with USMLE step 1 scores or not associated with board certification, however both of these samples were taken from the Uniformed Services University, which is the United States only federal medical school.^7^^,^^8^

A strength of our study is that it examines the relationship between the MCAT scores and licensing exams in a Canadian context rather than in the American setting. Canada’s medical school admissions processes, mandate, medical education system, and licensing exams are unique in the world. Therefore, variables such as MCAT scores, gender, age, GPA, and MMI may interact differently or demonstrate different associations with medical licensing exam performance than they do in the United States or other countries. A few older studies have examined Canadian data. Unlike our study, one group examined MCAT total score and MCAT-VR score, and found that both were significantly correlated with MCCQE Part I scores, however predictive ability, and the remaining MCAT sub-scores were not assessed.^13^ Another Canadian study, which had some similar findings to our study, concluded that the MCAT-VR score and the MCAT-BS score significantly correlated with MCCQE Part I scores.^12^ However, both of these studies were published more than a decade ago and as described earlier, a new MCCQE Part 1 minimum pass level and scoring procedures have been recently implemented. A more recent Canadian study by Roy et al. demonstrated a low degree of correlation between the MCCQE Part 1 and both MCAT and GPA, however, MCAT sub-scores were not assessed.^14^ Our study examines more current data, and provides evidence that is relevant to contemporary medical school admissions processes. Moreover, The Future of Medical Education in Canada (FMEC): A Collective Vision for MD Education, in 2010, described priority areas for Canadian medical schools to consider, including addressing community needs, medical leadership, and obligations to Indigenous people. This mandate and call to action has resulted in admissions processes frequently placing increased emphasis on traits and attributes, frequently non-academic, felt to be associated with success in these domains. Continuing to balance these non-academic selections criteria with factors that predict the ability to successfully complete licensing requirements and enter practice must not be forgotten in this complex climate.

Ultimately, medical students must successfully complete all licensing examinations prior to commencing clinical practice. Therefore, selecting applicants with the highest probability of meeting this objective, in conjunction with desirable non-academic attributes, should retain priority in the admissions selection process. The findings of this study suggest that it may be valuable to reconsider the importance of the science-oriented sections of the MCAT in student selection or implement a MCAT-BS cutoff score that applicants must meet for their file to be reviewed to increase the likelihood of passing the LMCC Part 1.

However, there are several factors that are important to consider before implementing these policy changes. Implementing MCAT cut-offs may disadvantage groups that tend to have lower scores such as applicants of low socio-economic status or with particular ethnic backgrounds.^9^^,^^15^ The MCAT may even have varying degrees of predictive validity across ethnic groups.^9^^,^^10^ As a result, incorporating an MCAT-BS cut-off into the admissions process may disadvantage certain groups of applicants and impede the school’s response to their own social accountability mandate. Additionally, non-academic skills that are highly desirable in physicians, including but not limited to communication, leadership and professionalism are not readily captured through standardized testing. Selecting students who will be able to move into clinical practice and represent society can be maximally optimized by using established predictive criteria while demonstrating the desirable non-academic skills. Admissions committees should ensure adequate education and training for individuals participating in file reviews and selections that reflect MCAT performance. If and how the MCAT is incorporated into the selections process in the Canadian context will likely continue to reflect the unique mission of each medical school.

Undergraduate GPA was not predictive of MCCQE Part 1 performance. It is conceivable that undergraduate GPA in programs of study relevant to medical school curricula (e.g. biology, biochemistry, etc.) may predict MCCQE performance. As well, the meaning of grades assigned from different institutions can vary considerably. Jones et al. (1983) convincingly demonstrated that similar grades from different undergraduate institutions can imply widely different levels of achievement.^16^ In our study, the MMI was not predictive of MCCQE Part 1 performance. The MMI is an assessment of both academic and non-academic attributes, including communication, empathy, collaboration, etc. which are seldom measured in a MCQ oriented examination such as the MCCQE Part 1.

It is unclear whether the same relationship exists between the MCAT-BS and performance in the MCCQE Part II, which consists of an objective structured clinical examination, and equally important to completing licensing certification. Future exploration of this relationship would be of interest.

A second limitation of our study relates to the version of MCAT we used to generate these results. In 2015, the Association of American Medical Colleges launched a new version of the MCAT. Although described as a “new exam,” the MCAT 2015 nonetheless involves significant overlap with the old MCAT in terms of question content. While the new exam consists of four sections rather than three, only the “Psychological, Social, and Biological Foundations of Behavior” tests material had not been previously tested. The other three sections, although renamed, cover similar material to the previous version. Most relevant to this study is that the largest degree of overlap occurs in the basic sciences (represented in the old Biological Sciences and Physical Sciences sections, where approximately 75% of questions related to biology, general chemistry, organic chemistry, and physics on the MCAT 2015 examination tests concepts that also appeared on the MCAT 1991 exam).^17^ Therefore, it is reasonable to think that the new MCAT 2015 sub-section will continue to be similarly predictive of licensing examination outcomes. There is no question, however, that application of our findings to admissions decisions in the MCAT 2015 era involves extrapolation from one test to another, and it will be important to confirm these findings using the MCAT 2015 itself. The CSM Admissions group were early adopters of the MCAT 2015, which will allow early evaluation of the predictive potential of the MCAT 2015 on the MCCQE Part 1 Exam in the near future.

### Conclusions

We have shown that the MCAT-BS predicts successful performance on the MCCQE Part 1, whereas the MCAT-PS, MCAT-VR, total MCAT score and undergraduate GPA were not predictive. Medical schools have a responsibility to balance the needs of the community with the academic mission of the faculty of medicine. Selecting applicants who will be successful in fulfilling this mission is imperative and multiple tools are necessary to achieve this goal. Positioning the MCAT and the respective sub-sections as an enabling factor to realizing this goal is likely to provide added value to the selections process.
